# Health system challenges to integration of mental health delivery in primary care in Kenya- perspectives of primary care health workers

**DOI:** 10.1186/1472-6963-13-368

**Published:** 2013-09-30

**Authors:** Rachel Jenkins, Caleb Othieno, Stephen Okeyo, Julyan Aruwa, James Kingora, Ben Jenkins

**Affiliations:** 1 Epidemiology and Mental Health Policy, WHO Collaborating Centre (Mental Health), Institute of Psychiatry, King’s College London, London, UK; 2 Department of Psychiatry, University of Nairobi, Nairobi, Kenya; 3 Great Lakes University, Kisumu, Kenya; 4 MONUSCO, Kisangani, The Democratic Republic of Congo; 5 Mental Health Programme, Kenya Medical Training College, Nairobi, Kenya; 6 Her Majesty’s Revenue Collection, London, UK; 7 WHO Collaborating Centre for Research and Training for Mental Health, Institute of Psychiatry, King’s College London, PO35, David Goldberg Centre, De Crespigny Park, SE5 8AF London, UK

**Keywords:** Health system challenges, Health sector reform, Mental health, Primary care, Kenya

## Abstract

**Background:**

Health system weaknesses in Africa are broadly well known, constraining progress on reducing the burden of both communicable and non-communicable disease (Afr Health Monitor, Special issue, 2011, 14-24), and the key challenges in leadership, governance, health workforce, medical products, vaccines and technologies, information, finance and service delivery have been well described (Int Arch Med, 2008, 1:27). This paper uses focus group methodology to explore health worker perspectives on the challenges posed to integration of mental health into primary care by generic health system weakness.

**Methods:**

Two ninety minute focus groups were conducted in Nyanza province, a poor agricultural region of Kenya, with 20 health workers drawn from a randomised controlled trial to evaluate the impact of a mental health training programme for primary care, 10 from the intervention group clinics where staff had received the training programme, and 10 health workers from the control group where staff had not received the training).

**Results:**

These focus group discussions suggested that there are a number of generic health system weaknesses in Kenya which impact on the ability of health workers to care for clients with mental health problems and to implement new skills acquired during a mental health continuing professional development training programmes. These weaknesses include the medicine supply, health management information system, district level supervision to primary care clinics, the lack of attention to mental health in the national health sector targets, and especially its absence in district level targets, which results in the exclusion of mental health from such district level supervision as exists, and the lack of awareness in the district management team about mental health. The lack of mental health coverage included in HIV training courses experienced by the health workers was also striking, as was the intensive focus during district supervision on HIV to the detriment of other health issues.

**Conclusion:**

Generic health system weaknesses in Kenya impact on efforts for horizontal integration of mental health into routine primary care practice, and greatly frustrate health worker efforts.

Improvement of medicine supplies, information systems, explicit inclusion of mental health in district level targets, management and supervision to primary care are likely to greatly improve primary care health worker effectiveness, and enable training programmes to be followed by better use in the field of newly acquired skills. A major lever for horizontal integration of mental health into the health system would be the inclusion of mental health in the national health sector reform strategy at community, primary care and district levels rather than just at the higher provincial and national levels, so that supportive supervision from the district level to primary care would become routine practice rather than very scarce activity.

**Trial registration:**

Trial registration ISRCTN 53515024

## Background

Health system weaknesses in Africa are considerable, constraining progress in meeting the Millennium Development Goals, including reducing the burden of both communicable and non-communicable disease [1]. The key challenges in leadership, governance, health workforce, medical products, vaccines and technologies, information, finance and service delivery have been well described [2]. In response, there have been thoughtful efforts to explore the core aspects of general health system strengthening [3], which has become a donor priority [4].

Mental disorders are major contributors to the global burden of disease, and there is increasing consensus that rather than remain an underfunded vertical programme, better outcomes could be achieved by horizontal integration into the health system. Indeed, since the declaration of Alma-Ata [5], integration of mental health into primary care has been a policy objective in a number of countries [6-8]. However, on the ground implementation in Africa has been difficult, and bedevilled by lack of political will, financial and human resource, and of research evaluation. There have been some evaluations of integration of specific interventions for single disorders or single client groups in low income countries, but not evaluation of comprehensive horizontal integration of all the usual mental disorders and client groups into routine primary care practice, within existing human and financial resources.

Since 2005, the Kenya Ministry of Health has conducted a programme to train 3000 primary health care staff from health centres and dispensaries across Kenya, in collaboration with the Kenya Medical Training College, Kenya Psychiatric Association and the World Health Organisation Collaborating Centre, Institute of Psychiatry, funded by Nuffield International Foundation and using a sustainable general health system approach [9,10]. In 2010 a pilot cluster randomised controlled trial was conducted to test the effect of this low cost training intervention (i) On the competencies of primary care staff to recognise mental disorders, treat and make appropriate referrals to the scarce specialist service; (ii) On recovery (improved health and social outcomes and quality of life) of clients.

The trial was conducted in a real clinical field setting, using local trainers and did not exert any special influence on the usual local availability of medicines, district supervision or local health management information systems. During the course of the trial, Kenya and especially Nyanza Province, experienced a severe shortage of medicines, and this was reflected in the research findings on medicine availability in the clinics participating in the trial. Nonetheless, the trial found significant improvement in the clients of the trained health workers in the intervention group compared with those in the control group (Jenkins R, Othieno C, Okeyo S, Kaseje D, Aweru J, Onyugi H, Bassett P, Torgerson D, Kauye F: Impact of mental health training on primary health care diagnostic skills and client recovery in Kenya - a controlled trial. Submitted). Therefore, at the request of the funder (the UK Department for International Development) we conducted focus groups with some health workers and clients from the trial to better understand their perspectives and experiences. Focus group methodology has been found to be an effective way to explore health worker and client views within health system contexts [11].

This paper is the third in a series reporting the results of the focus groups, the first presented the experiences and perspectives of the clients [12]; the second presented the experiences and perspectives of the health workers and their ability to understand, assess, and treat mental disorder [13]; and this third paper explores the generic health system difficulties encountered by health workers when trying to integrate mental health care into routine primary care practice.

## Methods

We contacted 20 health workers from 20 of the 100 clinics which had taken part in the RCT and invited them to take part in one of two ninety minute focus groups (10 primary care workers selected from the intervention group and 10 primary care workers selected from the control group). The primary care workers from the intervention group were placed in one focus group, and the primary care workers from the control group were placed in the second focus group. The health workers were male and female nurses or clinical officers. (Clinical officers have a 3 year medical training). We paid respondents’ transport and subsistence for the day they came for the interviews. No additional payments were made. There was no communication about the study between the different groups of participants before their respective focus groups were held. We were unable to conduct further focus groups with other health workers until data saturation was reached due to lack of further resources.

Ethical approval was obtained from Kings College London ethical committee and the University of Nairobi ethics committee. An explanatory information sheet was given to each health worker in English and Kiswahili; we explained verbally in English, Kiswahili and Luo to the participants the purpose of the meeting and asked for their individual consents. They were reassured of confidentiality and told that we wanted to learn from their experiences. We then assigned each participant an identification number. They were told that the conversations would be taped but they would not be identified by name.

The focus group discussions were held in August 2011, eight months after the end of the randomised controlled trial, during a residential two days in a small hotel at Chulaimbo, near Kisumu, which was easily accessible by all those invited. They were conducted by CO assisted by RJ, and each lasted 90–120 minutes. Discussions were conducted mainly in English, but also in the local language where participants could not understand English or found it easier to express themselves in Luo. A voice recording of all the sessions was made, and both RJ and JA took written notes which were used to help clarify the transcript material where necessary.

The focus group discussions were guided by the following exploratory questions, not all of which were eventually asked due to time constraints:

To start with could you tell us what you know about mental health?

What are your views concerning psychiatric disorders in this district, and in this health centre?

What are the common types of mental/psychiatric disorders encountered locally?

What is your experience in managing these cases?

Could you describe a specific case?

Are there any agencies involved in the management of the mentally ill? Please give examples.

In what way do they help?

Have you had any training in mental health? Please specify.

Did you attend the recently concluded training for primary health workers? Please describe your experience.

What were the positive things about the training?

What were the negative things about the training?

Did you benefit from the training?

How has it affected your practice?

Have the patients benefited in any way? Please give a specific example.

Please describe how you apply the knowledge and skills learnt in the training? If it is not applicable, please explain why.

Would you recommend the training to a colleague?

The recordings of the discussions were transcribed and translated into English where necessary. The voice recordings and the transcripts were analysed for common themes emerging in response to the guideline questions. Matrices were created to help facilitate the comparison of text across the different categories of informant.

## Results

11 health workers attended from the control group and 14 from the intervention group. This was more than the numbers invited, but these additional 5 attendees were included in the respective focus groups, and their statements are included in this analysis. This paper reports the results of the focus group discussions concerning health system issues which impact on the health workers ability to implement what they have learned during the mental health training programme.

### Community beliefs and pathways to care

The health workers in both intervention and control groups noted that since the community members attributed mental illness to demons they were more likely to take them to the church for prayers rather than to the medical centres:

*"They think it is bewitching*; *somebody has used some charm to make somebody berserk."* Health worker 8, control group.

They also noted that the patients taken to church for healing never recovered fully.

*"They feel it is demonic and they normally take them to the church to be prayed for but they don’t recover fully."* Health worker 10, control group.

Stigma was a major problem and they noted that mental illness was still more stigmatised than HIV/AIDS or leprosy. Patients were often neglected and were more likely to be taken to the health centres only when they were violent.

### Workload

The high number of clients who attend health centres each day make it difficult for the health worker to review each client adequately and discuss the problems comprehensively. Much time is taken up by other needs such as immunization and family planning. Some have solved this problem by opting to book clients who need counselling on the days when the workload is light:

"*One difficulty that I have personally is the number of clients that I am seeing. I am not able to take a lot of time with that client because the other clients are also there and the work load, other competing services like immunisation etc. So I tend to maybe give another date for this client to come."* Health worker, intervention group.

### Lack of supervision

In addition, applying the skills learned on the training course was not easy because the district medical team does not offer much support regarding mental health:

*"When they came round they had their own agenda and they seemed more interested in meeting the goals for other problems such as malaria, HIV, immunization. These have either been specifically funded or specific targets have been set at the national level."* Health worker, intervention group.

*"There is no supportive supervision as far as mental health is concerned … I think normally when these DPHNs (district public health nurses) are coming round they concentrate more on things like HIV, malaria, and immunisation. Nobody will talk about mental health/illness. So when you know they are coming, you also want to concentrate on what they will check and then at times, people tend to forget about the mental part of it."* Health worker 13, intervention group.

*"They come with their agenda."* Health worker, intervention group.

*"They are concerned about HIV."* Health worker, intervention group.

*"Them they are so much concerned about the areas dealing with HIV, immunisation and what have you and not mental health. So I think that is the area they are so much concerned with because they also want to achieve the targets. These are areas where some targets are. But you see in mental health, there is no target so nobody is interested."* Health worker 20, intervention group.

In cases where referrals were made to specialist care, the health workers found it difficult to obtain feedback about the client from the specialist. Additional problems faced by the health workers are poor compliance by patients with prescribed medication, and difficulty giving sustained follow up to clients.

### Multiple facility attendance

Clients often attend many health facilities, which militate’ against good consistent long term management of health problems:

*"Most of these clients…are in denial, and because of this, they would change the facilities, they would change the facilities each and every other time. So they are reviewed each and every other time. So when they come you may not be able to trace the background and find whether they have the illness or not."* Health worker 20, intervention group.

*"There’s like you are not helping me, you see, the features I am feeling are all these but you are not giving me this, so the best thing now is to drop you, to go to another health facility and start afresh."* Health worker 21, intervention group.

### Supply of medicines

A common challenge to both groups was the lack of appropriate medications in the health facilities. The drug supply was poor due to difficulties in using the "pull" system that required the health workers to order drug quantities for the clinics depending on clients’ needs and the clinic workload. Neither clients’ needs nor the workload in each clinic could be adequately captured by the health management information system since only two categories (mental illness and epilepsy) could be recorded on the sheet, and hence the single mental illness category could not be disaggregated into numbers of clients needing different types of psychotropic medicines such as antidepressants, anxiolytics, antipsychotics and anti-parkinsonian drugs. The basic psychotropic drugs available in some of the health centres included amitryptyline, phenobarbitone, chlorpromazine and diazepam. The latter was only available in injection form in some cases.

*"We only have valium.*" Health worker 1, control group.

*"We only have phenobarbitone.*" Health worker 8, control group.

*"In our facility we don’t have, we only have diazepam and phenobarbitone. We normally order but we have a push system where the KEMSA (Kenya Medical Supplies Agency) just bring us; and when we order they don’t bring at all."* Health worker 25, intervention group.

Often, the patients had to be sent to buy medication far from the facilities and had to cope with poor transport – in some cases only motorcycles could be used. Difficulties with transport also hindered referral to other facilities.

There was an excess of certain medicines such as panadol (paracetamol), oral contraceptives, and iodine. On the other hand, lack of certain essentials – gloves, gauze, syringes - meant that patients had to buy these themselves if necessary.

*"Sometimes even syringes are not there…you send the relatives to go and buy. And you can imagine you are in level 2 in interior Kenya and there is no place near to buy that syringe … you have to take a motorbike to buy syringes."* Health worker 10, control group.

Further, according to the health workers, in addition to the new pull system there is still a parallel residual push system for a small drug kit which goes to all clinics, but is not standardised. Sometimes it only contains a little quinine, a few other anti-malarial drugs, antibiotics and aspirin. This creates further confusion in the health workers’ minds about whether they are expected to order medicines or not:

*"Like in level 2 it is not a pull system, it is a push system up to now so you do not order. You are given what they have, they had planned for it."* Health worker 1, control group.

*"Concerning mental health, it is actually push as they are saying but in other sections like ARVs that is when we order and maybe for other opportunistic infections, but with mental health drugs we use the push system."* Health worker 3, control group.

The health workers had each evolved different methods of acquiring some medications.

### Tally sheet and Health Management Information System (HMIS)

Health workers emphasised the problem with existing morbidity tally sheets:

"In our monthly reports of morbidity it just mentions mental disorder and is not specific to what type of mental disorder."

The point was made that it is therefore impossible to use data from the tally sheet to order specific medicines in adequate quantities. Information on mental health needs to be enhanced at all levels.

### Lack of mental health targets in the NHSSP

The health workers in the focus groups also indicated that clear targets for mental health need to be established and monitored from the national to the community level. There is need to use protocols for managing common psychiatric disorders. Ongoing support, supervision and training on mental health are crucial. The patient summary sheets and the ordering system need to be revised to facilitate the drug supply to the community.

### Lack of resources

The health workers noted that physical investigations were difficult to carry out, aggravated by lack of resource in the clinics, poverty of the clients, and long distances to suitably equipped facilities.

### Management of violence

Difficulties experienced by the health workers also included the management of violent patients. One participant in the mental health training programme felt threatened by such patients:

*"The patient can come and has got a knife in his pocket and you do not know what he is carrying, there should be programmes especially for health workers."* Health worker 8, control group.

"The challenge I have been having with those with acute psychosis, sometimes they become so violent that when you are alone you can’t manage."

### Poor compliance with medicines

Poor compliance was also a problem. The participants thought that this was due to the different illness models used by the patients from that used by the health workers [7,14,15]:

*"You see without the follow up you may not be sure the drugs will be used because the patients already have their way of thinking about the disease and the community also has a way of thinking about the disease so may be when you gave them the drugs to take home, they may use or not use." Health worker* 2, control group.

### Need to embed and disseminate the training more widely

The health workers all felt that the training programme should continue to be rolled out, that those who had already been trained by the Nuffield funded mental health programme could do more to train their colleagues and the volunteer community health workers, and that the district health management team (DHMT) also need to be trained:

*"I feel the managers will also need to go through this training … that is the DHMT…the district health medical officers, the medical officers, the medical students … people are talking about other things like immunisation and antenatal …who is talking about mental illness? Nobody."* Health worker 20, intervention group.

The health workers emphasised the need for those who have been trained to train the community health workers (CHWs) in early detection and referral:

*"At level 1 we have this community strategy but when we go through it … there is very little information on mental health … the community health workers are the ones who can identify these people faster because they are with these people at the river, at the posho mill, everywhere so they may know some abnormal behaviour within the community first and then maybe it is tackled early because the more the diagnosis is made late the more dangerous it becomes to the community…*" Health worker 10, control group.

The health workers suggested the need for a mental health advocacy campaign at all levels, consideration of use of mobile phones for supervision and management protocols, online consultation with district health workers, more involvement and education of relatives. They emphasised the need for CHWs to include mental health in their routine tasks, and urged that mental health should be strengthened in the new basic training curriculum for all health staff.

The focus group discussions confirmed that no other training course in mental health had been offered to the health workers during the period of the study, apart from the KMTC programme to staff from the intervention clinics in the randomised controlled trial. However, a majority had attended a training course in one or more of the following: nutrition, maternal and child health, and HIV related courses -

*"I got trained on community and home based care for people living with HIV-AIDS. It did not touch anything on mental illness."* Health worker 8, control group.

## Discussion

This is the first qualitative examination of health worker perspectives on generic health system issues which impact on mental health clinical and administrative practice at the primary care level in Kenya. The focus groups were conducted in Nyanza province, Kenya, in the public sector primary care system, and the study findings are limited to these groups, although are likely to have wider relevance for Kenyan primary care as a whole. The health workers in both intervention and control groups discussed a number of health system issues impacting on their ability to care for clients, and health workers in the intervention group described how these factors influence their ability to implement their newly acquired skills from the training. These issues highlight a number of generic organisational implications for primary care functioning in Kenya, and for the integration of mental health into primary care, especially in relation to the medicine supply, ordering medicine, the health management information system, community education, referral and collaborative care, supervision and the national health sector strategic plan.

Most of the health workers experienced some difficulties with ordering and supplying medicine to clients. At the time of the study, the Kenya health system was in a transition between the old "push" system (whereby a basic quantity of medicines were routinely supplied to clinics in a kit) and the new "pull" system (whereby clinics have to specifically order what they need) of medication supply. The health workers were not clear about whether to order medicines, or how to order medicines, and many health workers were not even aware of the new "pull" system being introduced. Districts were at different stages in implementation of the new system, and not all the health workers had the required ordering forms. When health workers did order medicines, but received none, then in future they ceased from ordering them. Since some health centres had been referring most of the psychiatric cases, they had not previously used much psychotropic medicine and so they could not now base their orders for medicines on what they had previously used, thus setting up a vicious circle which currently remains unbroken. The summary sheet used by some of the health workers is designed to base ordering of medicines on the workload. But the actual workload could not be accurately recorded without proper diagnosis, and without a range of categories in the tally sheet. If few clinical cases of mental disorder are reported then the medicine supply will also be low, and if the medicine supply is low, then patients would continue to be referred to the district level since there were no medications available at health centre level. Therefore the new "pull" system of ordering medicines is not yet working well. It is also clear that some health centres are highly constrained by the lack of basic items such as gloves, syringes, gauze and iodine.

The health workers, especially those in the intervention group, were able to reach the community through talks organised by the community chiefs. More formal ways of imparting information on mental health through school talks and pamphlets could also be explored. More support is needed for the volunteer community health workers and the village chiefs who both have potentially influential roles in mental health. They could perhaps also undergo some basic training on how to recognise mental illness.

The perspectives of health workers on specific aspects of assessment, diagnosis and management of mental disorders were explored in an earlier article [13] which found differences between intervention and control groups in their attitudes, understanding and practice. However, by contrast in this article, we have not found major differences between intervention and control groups in their perspectives on health system challenges to integration of mental health into primary care, but rather a concerted view of the significant problems that impede delivery of good care, and which are discussed above.

The health workers who had undergone training had found the application of their new skills constrained by their high workload, inadequate supervision and lack of support from the district medical team, poor drug supply, the lack of clear mental health objectives and targets in the national health sector strategic plan, and hence the low priority accorded to mental health. Thus mental health was non-existent or very low on the agenda of the district and lower level health teams. There was only one psychiatrist in the whole of Nyanza province, and he was of course logistically not able to visit and support all the hundreds of primary health care facilities in the province.

If mental health were to be included as a target at health sector levels 1 (community), 2 (dispensary), and 3 (health centre) in the national health sector reform strategy, and progress monitored, such a mental health target would then encourage and indeed enable district public health nurses to supervise and support primary care clinics around mental health issues.

It is relevant to any discussion of health system strengthening in Africa, and in particular in relation to mental health, that account should be taken of the conceptual frameworks for mental health and the attitudes of the public, clients and health workers, and these have been explored in Kenya in earlier studies [7,14,15], as well as elsewhere. In the 1990s, a population survey found that the public subscribed to a biopsychosocial view of mental illness, which was compatible with Kenya’s mental health policy which was rooted in the concepts of Primary Health Care, as articulated at Alma Ata. However, the public did not at that time expect biopsychosocial care from the health services, but rather only the biological/pharmacological component, relying on other care providers for psychosocial management [15]. Interviews at that time with district level health workers (at level 4 in the health system), demonstrated that, despite their training in mental health care and their theoretical knowledge of the principles of Primary Health Care, they exhibited attitudes and practice in keeping with a more medical model of health care, emphasising pharmacological treatment and expecting psychiatric patients to conform to the standard Sick Role [15].

By contrast, in the focus groups reported here, conducted some fifteen years later, the primary care health workers (who operate at level 2 and level 3 in the health system) expressed concern that local community beliefs led to patients either being neglected and only taken to health centres when they were violent, and/or being taken first to church for healing, and only later to the health centre, thus losing much valuable time before effective treatment was instituted. We do not have equivalent contemporaneous data from level 4 health workers, but discussions during continuing professional development workshops with level 4 health workers (both psychiatric nurses and generic district public health nurses) supports the view that attitudes in both these groups of health workers are evolving to be much more supportive of the integration of mental health into primary care.

The perspectives of clients were also explored in a previous article [12], where it was noteworthy that clients in both intervention and control groups felt that mental health was an appropriate matter for primary health care. The clients in the intervention group had noticed and appreciated enhanced communication, diagnostic and counselling skills in their respective health workers. The clients in the control group argued strongly for better health worker- patient relationships, respect for patients, not to be blamed, abused and assaulted by health care staff, or even chased away by them, and they called for better mental health skills in the health workers.

In relation to the methodology of our study, we had originally designed our guide questions to explore the potential reasons for the improvement in health, disability and quality of life in the clients of the intervention clinics where staff had received mental health training, compared with those of the control clinics. Thus the questions were designed to elicit open ended discussion about mental health care issues rather than to specifically focus on generic health system challenges, and the information thus obtained has been reported elsewhere [12,13]. It is therefore noteworthy that the generic health system issues highlighted in this paper arose spontaneously in discussions with both the intervention group and the control group, and not in response to leading questions about specific health service problems. With the benefit of hindsight, we consider the list of guide questions remain helpful to assist primary care staff to reflect on mental health issues, although they were too many for a 90 minute focus group. In future research we consider it would be valuable to add further questions designed to explore these generic health system issues in more detail, although we consider the use of mental health questions a good starting point so that the health workers during the focus group are considering the health system from the perspective of the mental health of their clients. If future research of this kind were to be funded, we would reduce the numbers in each focus group to 5 to enable more opportunity for contribution by each individual, we would extend the duration from 90 to 180 minutes (with an interim break), and we would hold more focus groups until data saturation was reached.

Whilst we recognise that it is possible that there may be participant concern about taping of interviews, our participants did not express any such concerns, and as researchers we found the tapes an invaluable record, as often the verbal discussion proceeded too fast for comprehensive note-taking of the richness of the dialogue, and there was also the added complexity of language issues, as the discussion frequently switched between English, Kiswahili and Luo.

## Conclusion

There are a number of generic health system weaknesses in Kenya which impact on efforts for horizontal integration of mental health into routine primary care practice. Health system strengthening (especially improved supplies, information systems, supervision, and planning) is likely to enable enhanced health worker effectiveness in all clinical areas, including mental health. A major lever for horizontal integration of mental health into the health system would be the inclusion of mental health in the national health sector reform strategy at community, primary care and district levels rather than just at the higher provincial and national levels, so that supportive supervision from the district level to primary care would become routine practice rather than very scarce activity. Enhanced community education about mental health by primary care teams and their community health workers in liaison with local community leaders is a crucial adjunct to addressing these health system weaknesses.

## Competing interests

The authors declare that they have no competing interests.

## Authors’ contributions

RJ was responsible for overall design of the study, obtained funding, and co-led the focus groups with CO, wrote the first drafts of the introduction and discussion, and subsequent drafts of the overall paper. CO chaired the focus group discussions, supplied the transcription of data and the main analysis of findings, and wrote the first drafts of the methods and results. SO supervised the local implementation of the study design. JA coordinated the invitations of client and health workers, and took notes of the focus group discussions. JK managed the logistics of the research project. BJ advised on the specific content of the focus groups, and organised the audio taping of the discussions. All authors contributed to and approved the final version of the paper.

## Pre-publication history

The pre-publication history for this paper can be accessed here:

http://www.biomedcentral.com/1472-6963/13/368/prepub
